# Barriers to culturally competent caring practices for LGBTQI+ persons: experiences of primary healthcare nurses in Gauteng, South Africa

**DOI:** 10.3389/fpubh.2026.1764164

**Published:** 2026-02-20

**Authors:** George Johannes Nkabinde-Thamae, Charlene Downing

**Affiliations:** Department of Nursing, University of Johannesburg, Johannesburg, South Africa

**Keywords:** cultural competence, inclusivity, LGBTQI+, primary healthcare, professional nurses, South Africa

## Abstract

**Background:**

Nursing is grounded in caring and supported by professional and legislative frameworks that mandate equitable services. Despite this, nurses often face challenges in delivering culturally competent care to marginalized individuals. In South Africa, lesbian, gay, bisexual, transgender, queer, intersex, and other (LGBTQI+) persons continue to experience healthcare disparities when accessing primary healthcare (PHC) services.

**Objective:**

This study aimed to explore and describe the barriers that PHC nurses experience in providing culturally competent care to LGBTQI+ persons in Gauteng Province, South Africa.

**Methods:**

An exploratory qualitative design was employed. Using snowball sampling, two focus group interviews were conducted with professional nurses. Data were collected using a semi-structured interview guide and were analyzed thematically.

**Results:**

Nurses reported being inadequately prepared to provide culturally competent care to LGBTQI+ people. Personal and systemic factors constrained inclusive practice.

**Conclusion:**

The findings of this study underscore a pressing need to empower nurses through training, support, and systemic change, thereby fostering inclusive and culturally congruent care for LGBTQI+ individuals in South African primary healthcare facilities.

## 1 Introduction

Culturally competent care refers to professional nurses’ ability to effectively deliver healthcare services that meet the social and cultural needs of individual patients (1). Within this context, culturally competent care requires professional nurses to provide care that is grounded in scientific knowledge, embedded in respect, and demonstrated through acceptance and humility (2). Further, this concept is underpinned by Madeline Leininger’s Transcultural Nursing Theory, which promotes culturally competent care that is both acceptable and meaningful to individuals or specific communities (3, 4). This theoretical framework was appropriate for this study, as it focused on culturally competent and patient-centered care (5, 6). Further, Gonzalez, Sperandio, Mullen, and Tuazon (7) advise that, in addition to culturally competent caring practices, professional nurses should practice cultural humility, learn from their patients, and provide relevant nursing care.

Additionally, cultural humility promotes self-reflection and continuous learning, providing professional nurses with a clear lens through which to recognize and understand individual differences, to achieve the desired health outcomes (8, 9). Cultural humility seems to have a direct influence on culturally competent care, as it underscores the importance of caring for individuals despite the differences they might reflect, and the need for professional nurses to be empowered to provide care to individuals accessing healthcare facilities. However, despite the imperativeness of culturally competent care and cultural humility within the healthcare environment, professional nurses within this study appeared to struggle with these concepts, especially toward self-identified LGBTQI+ persons. Drawing from recent African studies, through a scoping review, it has been discovered that most healthcare institutions in Africa prioritize health for individuals identified as heteronormative, which excludes LGBTQI+ persons from having access to healthcare services (10). In support of the findings of this scoping review, Mkhize and Maharaj (11) concur that self-identified LGBTQI+ persons experience health inequalities, which promote inaccessibility to healthcare services they need, which sadly compromises their general health and overall wellbeing.

The inability of professional nurses to provide culturally competent care to LGBTQI+ persons is often reflected in these individuals’ experiences of discrimination, homophobia, and stigma (12). A similar study by Muwanguzi et al. (13) found that professional nurses appeared to be uncaring toward LGBTQI+ individuals, as reflected in the literature documenting experiences of discrimination, stigmatization, prejudice, homophobia, and verbal and physical abuse experienced by LGBTQI+ persons. In addition, LGBTQI persons perceived incidents of professional nurses using incorrect pronouns as uncaring, as it leads to misgendering, a sense of disrespect, and a lack of inclusivity (14).

Significant barriers hinder professional nurses from providing culturally competent care to self-identified LGBTQI+ persons, including limited confidence and knowledge deficiencies relating to the healthcare needs of LGBTQI+ persons (15). A study conducted in South Africa indicates that professional nurses, including nursing students, recognized that a deficit in knowledge and skills compromised the provision of quality care to LGBTQI+ persons (16). In Botswana, it was also discovered that professional nurses lack the knowledge and skills, and values to provide culturally competent care to LGBTQI+ persons, negatively affecting their mental well-being and overall health (17). Similarly, in Namibia, professional nurses seem to be unable to provide culturally competent caring practices to LGBTQI+ persons, as these individuals often express that professional nurses are uninformed about their healthcare needs, which frequently results in them experiencing poor healthcare services (18).

Professional nurses’ personal biases, religious beliefs, and practices also emerged as obstacles to providing culturally appropriate care to LGBTQI+ persons (19). Hafford-Letchfield et al. (20) state that when professional nurses allow their religious beliefs to negatively influence the care they provide to LGBTQI+ persons, this population is more likely to experience homophobic and stereotypical behavior when accessing healthcare services. Englund et al. (21) concur that such practices contribute to extensive discrimination, with some professional nurses even refusing to provide care to this population. The extent to which professional nurses allowed their religious practices to impact the care they provided to LGBTQI+ persons is illustrated in the study titled” ‘*He’s gay, he’s going to go to hell’: Negative nurse attitudes towards LGBTQ people on a UK hospital ward*.” Although this study was conducted within a hospital environment in the United Kingdom, its findings clearly demonstrate how professional nurses’ religious beliefs can negatively shape care practices and contribute to uncaring experiences for LGBTQI+ persons (22).

In addition, the healthcare environment itself hinders professional nurses from providing culturally appropriate care to LGBTQI+ persons. This is evidenced by a notable absence of unisex toilets and tailored resources – such as lubrication, finger gels, and hormonal treatment – which collectively promote a healthcare environment designed primarily for heterosexual persons and, in turn, directly exclude LGBTQI+ individuals (23). Literature globally indicates that approximately 65% of professional nurses exhibit prejudice toward LGBTI+ persons when they access healthcare institutions, which hinders nurses’ ability to provide culturally competent care to this population (24, 25). Thus, the findings of this study aim to raise awareness about the experiences of LGBTQI+ people in primary healthcare facilities, which may inform current and future policies and curriculum development, ultimately promoting caring practices that are inclusive, dignified, and equitable.

## 2 Materials and methods

### 2.1 Design

For this study, a qualitative, descriptive, and contextual approach was employed, which enabled the researchers to provide a detailed description of participants’ experiences in relation to the phenomenon under examination (26). In addition, this research design prompted the researchers to bracket their own views and beliefs as participants shared their experiences of providing culturally competent care in primary healthcare facilities (27).

### 2.2 Participants, setting, and data collection

The participants were recruited by the primary researcher through purposive sampling, using the snowball method. The snowball sampling method enabled the primary researcher to ask participants who consented and participated in the study to refer other relevant individuals who met the study’s criteria (28, 29). Moreover, Tornava et al. (30) found that the snowball method was suitable for research perceived as sensitive and that participants needed to trust the researchers. In addition to recruiting relevant participants, the researchers utilized social media platforms, including Facebook. The primary researcher’s contact information was provided to those interested in the study who met the study’s criteria. The inclusion criteria required participants to be employed at primary healthcare facilities in the Gauteng province, South Africa (City of Ekurhuleni, Sedibeng, Tshwane, City of Johannesburg, and Westrand). Malematja et al. (31) advise that primary healthcare facilities provide services such as maternal and child health, reproductive health, chronic disease management, and acute care. These healthcare services are provided to individuals at no cost by skilled professional nurses (32–34). In addition, participants had to have been employed at a primary healthcare facility for more than 2 years and to have provided healthcare services to LGBTQI+ persons within the past 6 months, to ensure that the experiences shared were current and meaningful. Data collection was conducted from 2 December 2023 to 15 March 2024, through two independent focus groups. FG1 consisted of seven (7) participants, and FG2 also consisted of seven (7) participants, yielding a total of 14 participants. Data saturation occurred when no new themes or subthemes emerged; thus, the two independent focus groups appeared adequate to meet the study’s objective, as the participants (professional nurses) were from all five districts in the Gauteng province. These focus groups were deemed the most suitable option compared to individual interviews, as they encouraged shared reflection on collective norms among professional nurses.

The demographic information of the participants is provided in Annexure A. The two focus groups were conducted in English by the primary researcher and transcribed verbatim, and all participants were fluent in spoken English. The focus groups lasted 60–90 min, and one central question was asked: “How do you experience providing care to LGBTQI+ persons?” This central question allowed the participants to share their own experiences and provided the researchers with deep insight into the phenomenon. In addition, communication skills, such as clarification, probing, silence, and questioning, enabled the primary researcher to ask follow-up questions, which assisted in developing the interview guide in Annexure B.

### 2.3 Data analysis

The researchers employed thematic analysis for this study (35). Wæraas (36) advises that thematic analysis is helpful for analyzing qualitative data, in which themes are refined systematically to derive meaning from the data. Ahmed et al. (37) share that Braun and Clarke’s six-phase analysis includes the following:

1. Familiarization with data

The primary researcher reviewed the field notes and all transcripts and listened to all voice recordings to make sense of the collected data.

1. Generating initial codes

This was a manual process in which the primary researcher systematically reviewed data collected from the two focus groups to identify and establish meaningful features.

1. Searching for themes

This stage required interpretive thinking, in which the primary researcher grouped similar themes. For this exercise, the primary researcher used mind maps.

1. Reviewing themes

The primary researcher reviewed the raw data to ensure the accuracy of the identified themes. During this process, specific themes were merged into one, divided, or even discarded, leading to theme repetition. This process promoted coherence and logic in relation to the identified themes and subthemes.

1. Defining and naming themes

All the themes were described, and the primary researcher reflected on their relevance to the research question. In addition, direct verbatim quotes were linked to themes to promote clarity and enhance relevance.

1. Writing the report

The primary researcher presented a detailed data analysis report to the secondary researcher, who served as the supervisor. This report included the themes, subthemes, and their interpretation. After the secondary research provided additional insights and prompted clarity-seeking questions, the identified themes and subthemes were discussed with an independent coder, an Associate Professor with extensive experience in qualitative research. The independent coder reviewed all the raw data to begin coding and consulted the primary researcher when clarification was required. In addition, regular meetings were held with the researchers and the independent coder, where any identified discrepancies were discussed and corrected, or any additional information was required, which ultimately led to the consensus or agreement on the final themes and sub-themes, as the independent coder had all the insight to determine the trustworthiness of the study and the provided information the researchers provided to the independent coder. This was done to promote transparency and prevent possible bias.

### 2.4 Ethical considerations

Ethical approval for this study was obtained from the Faculty of Health Sciences Research Ethics Committee at the University of Johannesburg (REC-1822-2022). In addition to gaining access to the various research sites (City of Ekurhuleni, Westrand, City of Johannesburg, Sedibeng, and Tshwane), the study had to be registered with the National Health Research Database (NHRD), and an approval reference was provided (GP_202211_014). The participants who met the inclusion criteria were invited to an information session, where the primary researcher shared the purpose of the intended research with them individually (38).

### 2.5 Trustworthiness

Trustworthiness in qualitative research promotes the authenticity and absolute truthfulness of the research findings (39). Trustworthiness was assessed using Lincoln and Guba’s criteria: credibility, transferability, dependability, confirmability, and authenticity (40).

## 3 Results

Based on the research information obtained during the two moderated focus groups, the following themes and subthemes were identified following the thematic analysis process (Table 1).

**Table 1:** Themes and subthemes.

Themes	Sub-themes
Theme 1: Lack of preparation	Inadequate knowledge, skills, and values to provide culturally competent caring practices to LGBTQI+ persons.
	Inadequate resources (finger gels, lubricants, preferred condoms, and hormonal tablets) to assist participants in providing culturally competent caring practices to LGBTQI+ persons.
Theme 2: Personal and Systemic Barriers	Their religious beliefs and practices had an impact on how they attempted to provide culturally appropriate care to LGBTQI+ persons. The infrastructure negatively contributed to the provision of culturally competent caring practices.

Self-Sourced by the authors.

### 3.1 Theme 1: lack of preparation

From the meaningful conversations during the two independent focus groups (FG1 = 7, and FG2 = 7, total 14 participants), it became evident to the researchers that the participants felt that they were not prepared to provide culturally competent caring practices to LGBTQI+ persons when they access primary health care facilities; it sounded as if the unpreparedness was related to the knowledge deficit. The following verbatim quotes give insight into the participants’ reality:

*“I feel like I’m not well educated, or rather empowered, when it comes to dealing with LGBTQ people; maybe a bit of education can help.” (Participant 3, FG 1).*

Other participants added:

*“I think we need education; I make mistakes often, because I rely on what I think is right or normal.” (Participant 4, FG 1).*

*“The gay community blames me for not knowing what happens in the gay community, their pronouns, and what the primary healthcare facilities need to provide to them, like their lubricants and safe condoms. It’s not fair to me, because no one has provided me with that knowledge.” (Participant 5, FG 2).*

*“I have all these qualifications, but none of them have prepared or empowered me on how to provide care to these people. I don’t even know what LGBTQI+ means, hence I say these people.” (Participant 6, FG 2).*

The unpreparedness to provide culturally competent care to LGBTQI+ individuals is clarified with the subtheme below.

#### 3.1.1 Subtheme 1.1: inadequate knowledge, skills, and values to provide culturally competent caring practices to LGBTQI+ persons

Participants expressed that, despite the different levels of training they received throughout their nursing careers, they felt unprepared to provide culturally competent caring practices to LGBTQI+ persons. They reported insufficient knowledge related to LGBTQI+ persons’ health needs, which hindered their ability to deliver culturally competent care.

The following verbatim quotes give more insight into this phenomenon:

*“Despite not being trained in the LGBTQI+ community and their healthcare needs, I often come across those who say they are transgender who need nursing care from me. I often find it difficult to provide care to a person who is transgender because I don’t know what to call them. I am even afraid to ask them certain questions because I would not want to ask the wrong questions and upset them, so I would say guidelines and policies should be developed to at least give us, the nurses, guidance on providing care to transgender individuals and other members of the gay community.” (Participant 7, FG 1).*

*“If a transgender women request hormonal tablets, how do I give him that because the available guidelines do not make provision for transgender women?” (Participant 5, FG 2).*

*“I do not have the knowledge, or skills, or perhaps not even the personal desire to provide care to the LGBTQI+ community.” (Participant 6, FG 2).*

*“This is a professional environment, and the nursing profession, how do I understand them, if the nursing institution where I trained did not include them in the nursing curriculum? How must I relate to, or understand, what they need? Some want to be called a female, whilst they clearly have male features, that’s visible.” (Participant 4, FG 1).*

The unpreparedness to provide culturally competent, caring practices to self-identified LGBTQI+ persons appears not to be linked solely to a knowledge deficiency stemming from a lack of formal or informal training; the subtheme below further provides insight into what contributes to this unpreparedness.

#### 3.1.2 Subtheme 1.2: inadequate resources to assist the participants in providing culturally competent caring practices for LGBTQI+ individuals

The participants shared that basic healthcare resources, such as condoms and lubricants, were lacking in healthcare facilities, and the available condoms were not what the LGBTQI+ persons desired, but were rather tailor-made for heterosexual individuals, which further promoted non-inclusivity and a non-affirming healthcare environment. The verbatim quotes give more insight.

*“It’s a bit difficult because, for example, let us say we get a lesbian couple with an STI, and then you tell them to use a condom; exactly which condom are you talking about because the condoms lesbian prefer are not available in this clinic, so are you saying to the patient use a condom but I cannot give you the condom, you should go to the shops and buy it?” (Participant 5, FG 2).*

*“…Sometimes, I don’t know what to say if a gay man asks me why we don’t have stronger condoms to give to the LGBTQI+ community; even lubricants are out of stock, that’s not all they ask for, sometimes they ask for a unisex toilet, which we don’t have …” (Participant 1, FG 1).*

*“I have never heard of finger gels. What do they even use them for? Where do we even order them?” (Participant 4, FG 1).*

*“I share the same sentiments as you; we heterosexuals only know of condoms, provided to a normal male and female.” (Participant 2, FG 1).*

### 3.2 Theme 2: personal and systemic barriers

In addition, the participants revealed that personal and systemic barriers negatively affected the care they provided to LGBTQI+ persons, which can be interpreted as internal and external factors. The following verbatim quotes illustrate how this theme was identified:

*“Despite the role of education and training not enabling me to provide appropriate care to gay, lesbians, transgenders, and others, it is the manner in which I was raised, and honestly also the entire setting of the environment, which is not my responsibility to change.” (Participant 4, FG 2).*

*“Yes, the nursing pledge, patients’ rights charter advocates that I, as a nurse, need to treat everyone with respect and dignity, but I am a Christian; there are certain compromises I just won’t be able to do.” (Participant 5, FG 2).*

*“A transgender will need certain treatment that is designed for females, how do I respond without looking or sounding homophobic or insensitive?” (Participant 3, FG 1).*

*“I cannot just change the infrastructure and create a toilet that is unisex, that’s beyond my duties and responsibilities as a nurse.” (Participant 2, FG 1).*

The verbatim quotes shared above are explicitly explained with the subtheme below.

#### 3.2.1 Subtheme 2.1: their religious beliefs and practices had an impact on how they attempted to provide culturally appropriate care to LGBTQI+ persons

The participants shared that they grew up within Christian homes, which embedded within them the idea that homosexuality is a sin, and only heterosexual norms are deemed acceptable. This philosophy appeared to have contributed to the care they provided to LGBTQI+ persons.

Two participants from the second focus group shared the following:

*“I think one of the contributing factors is religion. A lot of us grew up in Christian homes, and if you look at all the different religions, they are all against the LGB community.” (Participant 5, FG 2).*

*“My religion has an impact on how I say or do things; I find it different to provide compassionate care to those who say that they are gay or lesbian.” (Participant 3, FG 2).*

In addition, the verbatim quotes below gave insight into the extent to which the participants said religious beliefs and practices served as a barrier to providing culturally competent care to LGBTQI+ persons.

*“I don’t know how to react to a transgender woman that I can see is a man, but wants healthcare services that are only applicable to women. I am a Christian, and we were taught that God does not like people who sin.” (Participant 6, FG 1).*

*“Even if I wanted to, I would never accommodate them, not even by the way I address them, my religion only recognizes ‘Male’ or ‘Female’. If I do anything outside that, I would be sinning as well; the words gay or lesbian are not understood in the Christian family.” (Participant 5, FG 1).*

This sub-theme clearly demonstrated that primary healthcare nurses upheld their religious practices rather than adhering to the values of nursing, caring, ubuntu, and humanity, thereby promoting culturally competent caring practices, inclusivity, and affirming care for LGBTQI+ persons.

#### 3.2.2 Subtheme 2.2: the infrastructure negatively contributed to the provision of culturally competent caring practices

Moreover, the participants stated that infrastructure is not accommodating for LGBTQI+ persons, as evidenced by the fact that most formal or informal complaints received in primary healthcare facilities relate to the presence of only ‘Male’ or ‘Female’-labeled toilets, thus making the absence of a unisex toilet particularly notable.

*“Make a toilet that’s neither male nor female… That is the safer option.” (Participant 1, FG 2).*

*“There’s a toilet for people who are physically disabled, there’s female toilets, there’s male toilets, but there aren’t any unisex sexed toilets.” (Participant 2, FG 2).*

*“I did not realise how important a unisex toilet was, until recently, when we made a count of the number of complaints our facility has, which resulted in the LGBTQI+ community requesting toilets that accommodate them.” (Participant 1, FG 1).*

*“Sometimes there are fights in the toilets, because other patients, especially women, are not comfortable sharing toilets with transgender people.”* (Participant 2, FG 2).

This sub-theme suggests that participants were advocating for unisex toilets to promote inclusivity for LGBTQI+ people. The need for gender-neutral toilets appears to be an urgent need, as evidenced by the fact that physical fights even arose in primary healthcare facilities, contradicting the perception that healthcare facilities should be a safe space for all.

## 4 Discussion

This study aimed to examine the barriers that professional nurses experience in providing culturally competent care to self-identified LGBTQI+ persons when accessing primary healthcare facilities in Gauteng, South Africa. The results revealed various realities that affect primary healthcare nurses’ ability to provide appropriate care to these marginalized individuals. The findings of this study also highlighted the need to empower professional nurses regarding the importance of providing inclusive, respectful, and dignified care. This aligns with the positions of the American Academy of Nursing (41) and the International Council of Nurses (42), which advocate for equality of human rights in healthcare, including for LGBTQI+ persons.

### 4.1 Knowledge and skills gaps

The participants reported being unprepared to provide culturally appropriate care, attributing this to a lack of knowledge, skills, and values. This finding aligns with similar studies, which found that primary healthcare nurses have insufficient knowledge on how to provide culturally appropriate care to LGBTQI+ individuals (43). Inadequate knowledge, skills, and values among professional nurses regarding the healthcare needs of LGBTQI+ persons create significant barriers to the provision of appropriate healthcare services to this population (44). More than 30 independent studies have found that professional nurses are not adequately empowered to provide appropriate care to LGBTQI+ persons, which often leaves nurses feeling unprepared and contributes to ongoing healthcare disparities experienced by LGBTQI+ individuals (45). Furthermore, Klepper et al. (46) reveal that the few available textbooks contain insufficient or non-significant information to empower professional nurses to provide inclusive healthcare to LGBTQI+ people. There is a limited supply of teaching and learning material, as only three textbooks appear to include content relevant to LGBTQI+ persons, their healthcare needs, and general health disparities they experience (47).

In addition to the limited availability of textbooks that equip professional nurses to care for LGBTQI+ persons and address their healthcare needs, the current nursing curriculum also appears to lack relevant content (48). Even in instances where professional nurses have received formal or informal training to care for LGBTQI+ persons, it is still not adequate to provide culturally competent caring practices to these individuals (49, 50). To address the knowledge gap in providing culturally competent, caring practices to LGBTQI+ persons, McCann and Brown (51) state that the nursing curriculum should include content such as theoretical instruction, skills simulation, and practice-based learning, as well as relevant theories such as gender, queer, and social justice. These components will provide professional nurses with insight into the lived experiences of this population. The findings of this theme highlight the importance of empowering professional nurses to provide inclusive care, demonstrating the need to realign the current curriculum to address knowledge deficits professional nurses experience in providing care to LGBTQI+ persons.

### 4.2 Resource deficits

In addition to the knowledge and skills deficit, professional nurses stated that a barrier to the provision of culturally appropriate care was the unavailability of resources. The participants shared that essential resources needed in primary healthcare facilities were not available, which led them to view the general environment as not accommodating LGBTQI+ persons. This aligns with findings indicating that healthcare facilities do not prioritize the unique sexual health needs of LGBTQI+ people, resulting in the non-provision of imperative resources such as lubricants, finger gels, and preferred condoms (52). The inability of professional nurses to provide lubricants, finger gels, and condoms contributes to the marginalization of LGBTQI+ people (53). Thus, Fu et al. (53) advocate that the non-availability of these resources often denies LGBTQI+ persons access and communicates a message that healthcare organizations do not provide healthcare services for LGBTQI+ people. The non-availability of condoms and lubrication may be the rationale for the LGBTQI+ persons being more susceptible to infections such as HIV/AIDS (54). A recent study by Gyamerah et al. (55) affirms that LGBTQI+ people in South Africa face a high infection rate, which is exacerbated not only by the lack of condoms and lubricants but also by stigma and discrimination that limit access to healthcare facilities. To address the consequences of inadequate availability of suitable or preferred condoms for the LGBTQI+ community, governments globally should consider these needs and integrate relevant information into all relevant curricula (56).

### 4.3 Infrastructure deficits (systemic discrimination)

In addition to the identified barriers to providing culturally appropriate care, Seretlo, Smuts, and Mokgatle (57) note that healthcare providers recognize that healthcare institutions frequently fail to accommodate LGBTQI+ persons by not providing appropriate bathroom facilities. Colliver and Duffus (58) and Porta et al. (59) advocate that healthcare institutions should promote gender-neutral bathrooms to foster acceptability and inclusivity. The presence of gender-neutral toilets is essential to ensure that LGBTQI+ persons feel safe and comfortable, as opposed to using bathrooms designed exclusively for heterosexuals, where they may experience bullying and intimidation from heterosexual patients (60). James et al. (61) and Kosciw et al. (62) confirm that LGBTQI+ persons are harassed and attacked in heterosexual bathrooms. A study by McGuire, Okrey, Anderson, and Michaels (63) concludes that gender neutrality is imperative not only for LGBTQI+ persons but also for marginalized individuals such as people with disabilities. The findings of this subtheme demonstrate the importance of gender-neutral toilets. However, the researchers are mindful that some individuals might not be comfortable with gender-neutral toilets. Thus, the conclusion is that not all toilets should be gender-neutral, thereby giving all individuals the opportunity to choose which bathroom to use.

### 4.4 Religion (personal beliefs)

The participants shared with the researchers that they felt as if their belief systems had a negative impact on the care they provided to LGBTQI+ people. A recent study unveiled that professional nurses’ religious beliefs have an impact on the way they provide care to LGBTQI+ persons, which is normally embedded in discrimination and bias (64). Westwood, James, and Hafford-Letchfield (22) affirm that professional nurses confessed that, because of their religious beliefs and practices, they prefer not to engage with LGBTQI+ people. A quantitative study found that professional nurses perceived individuals classified as LGBTQI+ as having a mental illness and being possessed by evil spirits, resulting in these individuals not being accepted within healthcare environments (65). These findings are supported by Müller (66), where a patient stated, “As soon as I declared my sexuality, my physician perceived me as an ‘evil spirit’*”.* Another study, also within the South African context, shared a verbatim quote*, “I believe God created Adam and Eve, and that’s how it should be*” (67). Bumgardner et al. (68) advise that healthcare organizations need to raise awareness about how an individual’s religion can have a negative impact on the care they provide to LGBTQI+ persons. The findings of this theme indicate that professional nurses are not upholding the values and principles of the nursing profession, such as the patient rights charter, *batho pele* principles, caring or *ubuntu*, but rather their own personal beliefs.

## 5 Reflexivity statement

Reflexivity was maintained throughout the entire research. Data were collected by the primary researcher, a PhD candidate from an esteemed university. Furthermore, the primary researcher has adequate knowledge and skills in conducting individual interviews and continues to engage in activities to promote rigorous, transparent, and constructive research. The primary researcher participates in peer reviews (local and international journals). Moreover, the secondary researcher served as a supervisor, during which all recorded interviews were listened to, and regular meetings were held between the researchers. In addition, the secondary researcher is a professor (full) in nursing science and has a respectable reputation within the nursing profession. In addition, the independent moderator during independent focus groups prevented bias or coercion of participants. Lastly, an independent coder was hired to analyze the data. These processes allowed the researchers to bracket their own perceptions of the phenomenon, enabling the participants to freely express their realities and providing truthful findings for the study.

## 6 Implications for policy

The findings highlight the urgent need for healthcare policies that explicitly recognize affirming and culturally competent caring for self-identified LGBTQI+ persons as a core nursing responsibility. All institutional and regulatory policies should incorporate LGBTQI+-affirming care behaviors into CPD (Continuing Professional Development) requirements, professional standards, and quality assurance frameworks. Embedding caring indicators related to dignity, respect, and psychological safety within performance appraisal and patient experience metrics may support consistent, equitable care and reduce disparities that self-identified LGBTI+ persons experience in healthcare facilities.

## 7 Implications for nursing curriculum reform

The study highlights the importance of moving beyond the implicit assumption that caring competencies naturally develop through clinical exposure. Nursing curricula should intentionally integrate LGBTQI+ health content grounded in caring theory, reflective practice, and ethical formation. Longitudinal inclusion of inclusive communication, bias awareness, and relational caring skills across undergraduate, postgraduate, and CPD programs may better prepare professional nurses to deliver affirming, person-centered care to self-identified LGBTQI+ persons.

## 8 Future research priorities

Future research should focus on evaluating the sustainability and long-term impact of care-focused educational interventions on nursing practice and self-identified LGBTQI+ persons’ experiences of care in healthcare facilities. Longitudinal and mixed-methods studies exploring patient-reported outcomes, nurse moral resilience, and organizational support for inclusive care are needed. Comparative research across healthcare sectors may further illuminate contextual enablers and barriers to implementing caring practices for LGBTQI+ persons.

## 9 Conclusion

Professional nurses employed in primary healthcare facilities in Gauteng, South Africa, face multiple barriers that hinder the provision of culturally appropriate care to self-identified LGBTQI+ individuals. Therefore, relevant teaching and learning strategies should be prioritized through curriculum reform to equip current and future professional nurses with the necessary knowledge and skills to deliver dignified, respectful care. Additionally, this study’s findings highlight the need for systematic accountability and for realigning national training modules through curriculum reform.

### 9.1 Strengths of the study

This study was conducted to examine the barriers professional nurses experience when providing care to self-identified LGBTQI+ persons. Based on the available literature, this appeared to be the first study of its kind in the context of primary healthcare facilities, providing significant insight into the identified barriers and their negative impact on the care nurses provided. Moreover, the findings of this study may be generalized to other contexts beyond primary healthcare facilities, as the researchers provided detailed information on the research setting, participants, and the methodology used throughout the study.

### 9.2 Limitations of the study

This study was conducted only in primary healthcare facilities in Gauteng, which may have imposed geographical limitations. Additionally, despite employing a qualitative methodology, this study yielded relevant results. However, a quantitative methodology might have reached a larger number of participants, yielding more findings, as a larger sample size would have allowed for greater statistical power.

## Data Availability

The raw data supporting the conclusions of this article will be made available by the authors, without undue reservation.
