# Supplementary material for: Barriers to culturally competent caring practices for LGBTQI+ persons: experiences of primary healthcare nurses in Gauteng, South Africa
Source: Front Public Health. 2026 Feb 20;14:1764164. doi: 10.3389/fpubh.2026.1764164 (PMC12963211; doi:10.3389/fpubh.2026.1764164)
Supplement: Supplementary file 1 [file Supplementary_file_1.docx]

**Annexture A: Demographics of the participants**

| Focus Group information | Age | Gender | Qualifications | Years of Service |
| --- | --- | --- | --- | --- |
| P1 FG 1 | 43 | Female | BCur Degree Nursing | 11 years in a primary healthcare clinic |
| P2 FG1 | 39 | Male | Diploma in General Nursing and Midwifery | 13 years in a primary healthcare clinic |
| P3 FG1 | 55 | Female | BCur Degree in Nursing, Specialty in Primary Healthcare | 20 years in a primary healthcare clinic |
| P4 FG1 | 29 | Female | Diploma in General Nursing Science and Occupational Health Nursing Science. Currently busy with a Diploma in Primary Healthcare | 7 years in a primary healthcare clinic |
| P5 FG1 | 54 | Female | BCur Degree Nursing, Diploma in Primary Healthcare, Occupational Health Nursing Science. | 23 years in a primary healthcare clinic |
| P6 FG1 | 33 | Female | BCur Degree Nursing | 5 years in a primary healthcare clinic |
| P7 FG1 | 49 | Female | BCur Degree Nursing | 9 years in a primary healthcare clinic |
| P1 FG2 | 39 | Female | Diploma in General Nursing and Midwifery | 11 years in a primary healthcare clinic |
| P2 FG2 | 44 | Female | Diploma in General Nursing and Midwifery | 20 years in a primary healthcare clinic |
| P3 FG2 | 25 | Female | BCur Degree Nursing | 7 years in a primary healthcare clinic |
| P4 FG2 | 33 | Female | BCur Degree Nursing | 6 years in a primary healthcare clinic |
| P5 FG2 | 56 | Female | BCur Degree Nursing; Diploma in Primary Healthcare, Occupational Health Nursing Science. | 30 years in a primary healthcare clinic |
| P6 FG2 | 44 | Female | General Nursing Science, Diploma in Midwifery, Diploma in Primary Health Care | 14 years in a primary healthcare clinic |
| P7 FG2 | 22 | Female | BCur Degree Nursing, Diploma in Primary Healthcare | 4 years in a primary healthcare clinic |

**(**Self-sourced by the authors)

**Annexture B, Interview Guide**

| - What is your understanding of culturally competent caring practices |
| --- |
| - Can you describe how culturally competent caring practices assist in providing dignified and respectful healthcare services to self-identified LGBTQI+ persons? |
| - How will adequate knowledge, skills, and values assist in the provision of culturally competent caring practices to self-identified LGBTQI+ people? |
| - What internal and external barriers have you experienced in providing culturally competent caring practices to self-identified LGBTQI+ people? |
| - What specific resources are available with primary healthcare facilities that promote culturally competent care practices |
| - What specific resources are needed to promote culturally competent caring practices towards LGBTQI+ persons |
| - How do personal and systematic barriers impact your ability to provide culturally competent caring practices to LGBTQI+ people? |

(Self-sourced by the authors)
